# All That Beeps is Not Urgent: Hospitalist and Nurse Concordance of Assigning Priority Classification to Pages

**DOI:** 10.1007/s11606-024-08962-w

**Published:** 2024-09-03

**Authors:** Sarah J. Flynn, Esther Y. Hsiang, Molly A. Kantor, Madhu N. Rao, Raman Khanna

**Affiliations:** 1 Division of Hospital Medicine, Department of Medicine, University of California, San Francisco, San Francisco, CA USA; 2 Division of Endocrinology and Metabolism, Department of Medicine, University of California, San Francisco, San Francisco, CA USA; 3 Division of Clinical Informatics and Digital Transformation, Department of Medicine, University of California, San Francisco, San Francisco, CA USA

## INTRODUCTION

Miscommunication contributes to medical errors during hospital admissions.^1,2^ Within the inpatient setting, interdisciplinary communication between nurses and providers often occurs via messages sent as alphanumeric (text) pages. Prior studies have found that many messages sent as pages are non-urgent and often lack indicators of response urgency.^3–5^ With the adoption of secure messaging systems to allow for bi-directional text messaging, the volume of direct messages received by inpatient providers may be substantially increasing.^6^ Therefore, accurately triaging message urgency is crucial.

Our institution implemented a tiered text paging system which encourages (but does not require) senders to assign priority labels of “FYI” (i.e. informational, no action required), “ACTION” (i.e. action required, non-urgently), or “911” (i.e. urgent action required) to messages. In this study, we investigated the frequency with which pages sent from nurses to hospitalists included priority labeling, the proportion of informational messages sent during nightshifts, and the concordance between hospitalist and nurse priority classifications.

## METHODS

We analyzed all pages sent through an electronic paging system from nurses to hospitalists on the direct care medicine service at a large, urban academic medical center over a 5-week period. We recorded how many of these pages included labels of “FYI”, “ACTION”, or “911”. We also analyzed whether priority classification differed between daytime hours (6:00a.m. through 7:59p.m.) and nighttime hours (8:00p.m. through 5:59a.m.). To evaluate concordance between priority classifications, two hospitalists (SF, EH) reviewed a randomized subset of pages masked to the original priority label and independently assigned labels to these messages. A third, senior hospitalist (MK) served as a tiebreaker in the event of disagreement between the two reviewers. Message priority classifications by hospitalists were then compared to the original label indicating message priority as chosen by the sending nurse. This study was approved by the University of California, San Francisco Institutional Review Board.

## RESULTS

From October 1, 2023 to November 9, 2023, the average daily census on the direct care hospital medicine service was 75.3 patients for 9 hospitalist teams. Over this time period, nurses sent 7,211 pages to hospitalists through the electronic paging system; of these, 18 messages were labeled “911”, 3,831 messages were labeled “ACTION”, 2,710 messages were labeled “FYI”, and 652 messages were unlabeled. Nurses sent fewer pages at night, but the priority labeling distribution was similar, including for FYI pages (37.9% day, 36.8% night; *p* value = 0.38) (Table 1).

**Table 1:** Proportion of Priority Labels of Messages Sent by Nurses at Night Vs. During the Day

Priority Label	Daytime (percent of total)^*^	Nighttime (percent of total)^*^
911	14 (0.3%)	4 (0.2%)^†^
ACTION	2,825 (52.6%)	1,006 (54.7%)^†^
FYI	2,033 (37.9%)	677 (36.8%)^†^
No Label	499 (9.3%)	153 (8.3%)^†^
TOTAL	5,371	1,840

Priority Label – original label, as applied by nurses at the time the message was sent

^*^Daytime = between the hours of 6:00a.m. and 7:59p.m. Nighttime = between the hours of 8:00p.m. and 5:59a.m

^†^*p* value by Χ^2^ test across all categories = 0.38

A convenience sample of 250 messages masked to their original priority labels were reviewed. The two hospitalist reviewers were outstanding in their agreement about priority classification assignments (kappa 0.80, p < 0.0001). The senior hospitalist reviewer adjudicated 26 discrepancies. The final hospitalist priority classification and the nursing priority classification exhibited moderate agreement (kappa 0.52, p < 0.0001). Hospitalists were more likely to label messages as “ACTION” than either “911” or “FYI,” and a small number of pages (n = 4) labeled by nursing as “911” were labeled by hospitalists as “FYI” (Table 2).

**Table 2:**
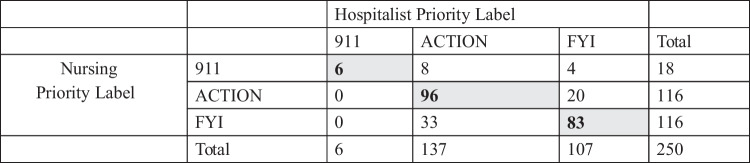
Concordance of Page Priority Classifications between Hospitalists and Nurses

Nursing Priority Label: label as applied by nursing when message was originally sent

Hospitalist Priority Label: label as applied by hospitalist reviewers

Gray cell with bold text indicates concordant labeling

## DISCUSSION

In our single institution study, a tiered paging priority labeling system had excellent adherence, with the vast majority of messages (91%) from nurses to hospitalists labeled by message priority. Consistent with past literature, our findings show that a considerable proportion of messages (37%) were informational in nature and non-urgent, including those sent at night.^3–5^

Hospitalist reviewers moderately agreed with the original priority label, with a tendency to consider more messages actionable than those labeled by nurses. This discordance may relate to variable interpretations of actionability and message urgency, as well as patient-specific clinical contexts being unknown to the masked reviewers. Improving the alignment of paging priority labeling between disciplines and standardizing messaging practices is especially important overnight, when provider staffing is often significantly reduced. With lower staffing levels, providers need to prioritize responding to the most urgent clinical needs. If providers are overwhelmed with informational pages, there is a risk that they may not address more clinically urgent messages which may contribute to poor outcomes or patient safety events.

Our study was limited to a single hospital medicine service at a single center and had a small sample size. Nonetheless, it suggests opportunities to better standardize communication practices, including the management of nighttime informational pages, and to improve alignment of paging priority labeling between disciplines to enhance communication between physicians and nurses and decrease the potential for overlooking pages that need urgent action.
